# Long-term use of dual orexin receptor antagonists for the treatment of insomnia: a systematic review and meta-analysis

**DOI:** 10.1055/s-0046-1827049

**Published:** 2026-08-13

**Authors:** Luis Miguel Moraes Araujo, Arthur Duarte de Sousa, Camila de Carvalho Vieira, João Pedro Pimentel Abreu, Cristiane Fiquene Conti, Melaine Mont'Alverne Lawall Silva, Marcio Moyses de Oliveira

**Affiliations:** 1 Coordenação do Curso de Medicina, Universidade Federal do Maranhão, São Luis MA, Brazil.; 2 Coordenação Especial de Morfologia (CEMOR), Universidade Federal do Maranhão, São Luis MA, Brazil.

**Keywords:** Orexin Receptor Antagonists, Sleep Initiation and Maintenance Disorders, Systematic Review

## Abstract

**Background:**

Insomnia is a prevalent disorder associated with impaired daytime functioning and reduced quality of life. Dual orexin receptor antagonists (DORAs) have shown favorable short-term efficacy and safety profiles. However, evidence regarding their long-term (≥ 6 months) effects remains limited.

**Objective:**

To synthesize the available evidence on prolonged DORA use in adults with chronic insomnia.

**Methods:**

We conducted a systematic review and meta-analysis of randomized controlled trial. Adults with primary insomnia treated with DORAs (suvorexant, lemborexant, or daridorexant) approved by the United States Food and Drug Administration (FDA) for ≥ 6 months were included. The outcomes included patient-reported sleep parameters, adverse events, and treatment discontinuation.

**Results:**

We included 6 randomized controlled trials comprising 3,546 participants. After 6 and 12 months, DORAs improved subjective sleep parameters of efficacy. Overall, DORAs showed adverse events similar to those of placebo at 6 months, but higher rates at 12 months. The overall rate of discontinuation due to adverse events did not differ significantly, although higher doses of certain DORAs increased the discontinuation rate at 6 months.

**Conclusion:**

In chronic insomnia, DORAs provide sustained improvements in key subjective sleep outcomes, with overall acceptable safety and tolerability profiles. Higher doses may be less well tolerated. Additional long-term randomized trials are needed to better define the efficacy and safety of DORAs in clinical practice.

## INTRODUCTION

Insomnia is a multifactorial sleep disorder characterized by difficulty initiating or maintaining sleep, leading to impaired rest and daytime functioning. Common symptoms include fatigue, irritability, and concentration problems, with significant social, occupational, and quality-of-life consequences. 1 The condition may occur as a primary disorder or in association with comorbidities such as obstructive sleep apnea and restless legs syndrome. 2 The risk factors include older age, depression, chronic illnesses, and socioeconomic vulnerability. 3

Epidemiological data indicate that 10 to 40% of adults experience clinically-relevant insomnia symptoms, 3 4 with prevalence reaching 19.6% in older adults 5 and being higher among women. 4 Longitudinal studies 6 show that insomnia often becomes chronic, persisting for years. Beyond the individual burden, it contributes to productivity loss, increased accident risk, and higher healthcare costs. 7

From a neurobiological perspective, the orexin system plays a central role in sleep–wake regulation. Orexins A and B, which are produced in the lateral hypothalamus, act on orexin receptor 1 (OX1R) and orexin receptor 2 (OX2R) to promote wakefulness. Dysregulation of this system is linked to insomnia and other neuropsychiatric conditions. 2 8

This knowledge has driven the development of dual orexin receptor antagonists (DORAs), which block OX1R and OX2R. Clinical trials 8 9 suggest they improve sleep, with fewer adverse effects on sleep architecture, lower dependence risk, and no rebound insomnia compared with traditional hypnotics. Since 2014, suvorexant, lemborexant, and daridorexant have been approved by the United States Food and Drug Administration (FDA), showing sustained efficacy and favorable safety profiles. 10 11 12 13

However, evidence on long-term (≥ 6 months) efficacy and safety remains limited and heterogeneous. Therefore, the current meta-analysis aims to critically synthesize the available data on prolonged DORA use in chronic insomnia, providing evidence to guide clinical practice and therapeutic decisions.

## METHODS

The present review was prospectively registered in the International Prospective Register of Systematic Reviews (PROSPERO; under CRD42024604300). The study was designed and reported in accordance with the 2020 Preferred Reporting Items for Systematic Reviews and Meta-Analyses (PRISMA) statement. 14

### Eligibility criteria

We included randomized controlled trials (RCTs) with no language restrictions. Eligible participants were adults (≥ 18 years) diagnosed with primary insomnia according to standardized diagnostic criteria, including the *Diagnostic and Statistical Manual of Mental Disorders, Fourth Edition, Text Revision* (DSM-IV-TR), 15 the *Diagnostic and Statistical Manual of Mental Disorders, Fifth Edition* (DSM-5), 16 the *ICD-10 Classification of Mental and Behavioural Disorders: Diagnostic Criteria for Research* (ICD-10-DCR), 17 *International Classification of Diseases, Eleventh Revision* (ICD-11) 18 or the *International Classification of Sleep Disorders—Third Edition* (ICSD-3). 19 Studies were excluded if they involved children or adolescents (aged < 18 years), patients with comorbid sleep disorders, and individuals with a history of complex sleep-related behaviors, unstable psychiatric disorders, or alcohol or substance abuse.

The eligible interventions were FDA-approved DORAs, including suvorexant, lemborexant, and daridorexant. The comparators included placebo, other DORAs, or active pharmacological agents such as benzodiazepines, Z-drugs, melatonin agonists, antidepressants, or antihistamines. The primary outcomes were patient-reported measures of insomnia: subjective time to sleep onset (sTSO), subjective wake after sleep onset (sWASO), subjective sleep efficiency (sSE), subjective total sleep time (sTST), and the total score on the Insomnia Daytime Symptoms and Impacts Questionnaire (IDSIQ). The secondary outcomes were safety and tolerability, including the occurrence of any adverse event (AE) that emerged or worsened after treatment initiation, serious adverse events (SAEs), and AEs leading to discontinuation.

### Search strategy and study selection

Two independent reviewers conducted a systematic search on the PubMed, Embase, SciELO, LILACS, and the Cochrane Central Register of Controlled Trials (CENTRAL) databases, from inception to 31 July 2025. The search strategy included controlled vocabulary and keywords related to DORAs and insomnia, combining generic and drug-specific terms. For example, one of the search strings used was: ( *orexin receptor antagonist* OR *orexin receptor antagonists* OR *orexin* ) AND ( *insomnia* OR *chronic insomnia* OR *sleep disturbance* OR *sleep disorder* OR *sleep disorders* ). Additional searches included specific drugs combined with insomnia-related terms ( *daridorexant* OR *lemborexant* OR *suvorexant* ).

All records were screened using the Rayyan (Rayyan Systems Inc.) systematic review software, 20 and data extraction was performed independently by two authors using standardized forms in Google Docs (Alphabet Inc.). Discrepancies were resolved through discussion, and unresolved conflicts were adjudicated by a third reviewer.

### Risk of bias assessment

Risk of bias was assessed using the Risk of Bias 2 (RoB 2; The Cochrane Collaboration) tool. 21 Each study was evaluated across five domains: randomization process, deviations from intended interventions, missing outcome data, measurement of outcomes, and selection of reported results. The overall risk of bias for each study was categorized as *low* , *some concerns* , or *high* . Any disagreements between reviewers were resolved by consensus or by a third author, if required. The certainty of the evidence for each outcome was planned to be assessed using the Grading of Recommendations, Assessment, Development, and Evaluations (GRADE) approach, 22 considering study limitations, inconsistency, indirectness, imprecision, and potential publication bias.

### Data synthesis and statistical analysis

Pairwise meta-analyses were performed using the Review Manager (RevMan; The Cochrane Collaboration) software, version 5.3. For continuous efficacy outcomes, results were expressed as mean difference (MD), while, for dichotomous safety outcomes, risk ratios (RRs) with 95%CIs were calculated. The analyses were conducted using a random-effects model to account for variability among studies. Heterogeneity was assessed using the I ^2^ statistic, with values < 30% considered *low* , between 30 and 50%, *moderate* , and > 50%, *substantial heterogeneity* .

Additionally, a network meta-analysis (NMA) was performed using the NMAstudio (free and open source), 23 an online platform. **Supplementary Material** (available at ( https://www.arquivosdeneuropsiquiatria.org/wp-content/uploads/2026/04/ANP-2026.0054-Supplementary-Material.docx ) Figure S1 (online only) shows the network relationship involving the various interventions, in which the nodes represent interventions and the lines represent direct trial comparisons. Treatment efficacy, safety, and tolerability were estimated by integrating direct and indirect evidence, expressed as MD for continuous outcomes and as risk ratio for dichotomous outcomes, with 95%CIs.

## RESULTS

**Figure 1:**
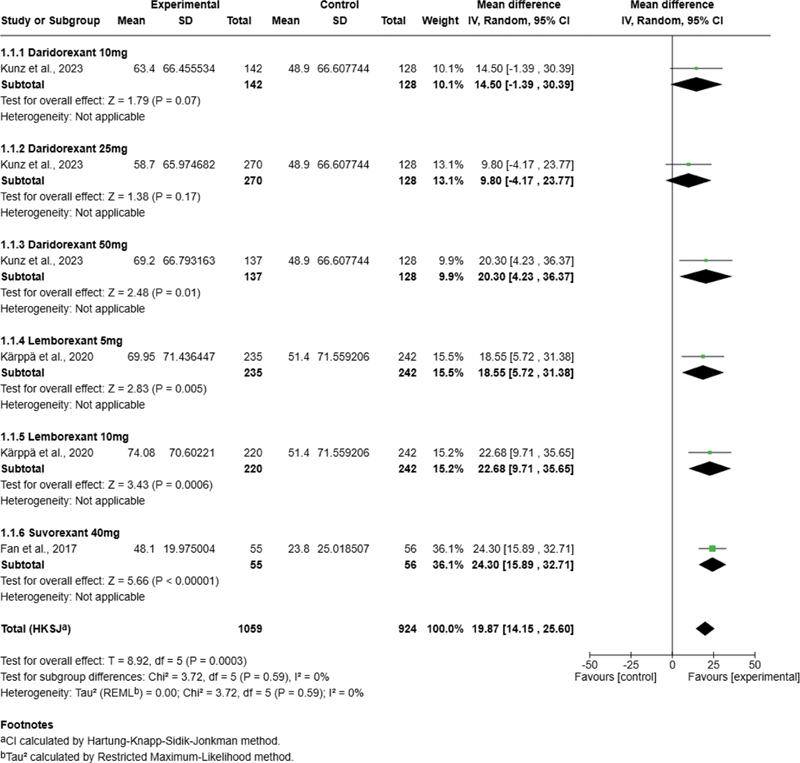
Efficacy of different dosages in the dual orexin receptor antagonists (DORAs) groups and the placebo group at 6 months: Subjective total sleep time (sTST).

From the database search, 9,929 articles were identified ( Figure 1 ). Of these, 5,959 were duplicates, and 3,951 were excluded after screening titles and abstracts, leaving 19 articles for full-text review. Subsequently, 13 studies were excluded, and the reasons are presented in **Supplementary Material Table S1** (online only). Ultimately, 6 RCTs 11 24 25 26 27 28 met the eligibility criteria and were included in the present review. None directly compared different insomnia drugs, as all evaluated DORAs versus placebo. Translation was not required, as no non-English manuscripts were included in the analysis. The main characteristics of the included studies are summarized in **Supplementary Material Table S2** (online only).

In total, 3,546 participants were included in the analyses. The sample from the study by Yardley et al. 24 was not counted here, as it reported results from the second phase of the Long-term Study of Lemborexant in Insomnia Disorder (SUNRISE 2) trial (≥ 6 months), involving the same cohort as in the study by Kärppä et al., 25 but without a placebo comparator. Three trials evaluated suvorexant (SUV): 2 studies 11 26 used 40 mg in participants aged < 65 years and 30 mg in those aged ≥ 65 years, one of those 11 also assessed 20 mg/15 mg (nonelderly/elderly), and another study 27 tested only the 40-mg dose, all compared with placebo. Two studies 24 25 reported different phases of the same trial evaluating lemborexant (LEM) 5 and 10 mg versus placebo, one 25 with results up to 6 months, and the other, 24 up to 12 months. Finally, one trial 28 tested daridorexant (DAR) at doses of 10, 25, and 50 mg versus placebo for 12 months.

### Risk of bias

Risk of bias was assessed using the ROB 2 tool. The efficacy outcomes from the studies by Kärppä et al. 25 and Yardley et al. 24 were judged as presenting a *high risk of bias* due to missing outcome data, and Fan et al. 27 raised *some concerns* regarding the selection of reported results. All other studies were judged as presenting a *low risk of bias* for the assessed domains: randomization process, deviations from intended interventions, missing outcome data, outcome measurement, and selection of reported results. Overall, most included trials were at a low risk of bias ( **Supplementary Material Figure S2** ). The certainty of the evidence according to the GRADE approach for each outcome is described in **Supplementary Material Table S3** (online only).

### Efficacy

All efficacy outcomes were synthesized using MD based on least-square mean (LSM) change from baseline, as reported in the original trials. Detailed results for each outcome are presented in Table 1 .

**Table 1:** Summary and effects sizes from the pair-wise meta-analysis of efficacy outcomes; from all trials using random-effects models at month 6

Outcome	Number of trials contributing to the meta-analysis	Number of participants contributing to the meta-analysisª	MD (95%CI)	*p* -value	I ^2^ (%)
Month 6					
1 sTST	2	1,485	19.87 (14.15–25.60)	0.0003	0
1.1 Daridorexant	1	677	14.37 (1.19–27.56)	0.04	0
1.2 Lemborexant	1	697	20.59 (-5.64–46.83)	0.06	0
1.3 Suvorexant	1	111	24.30 (15.89–32.71)	< 0.00001	N/A
2 sTSO	2	834	-12.69 (-19.78 to -5.60)	0.02	0
2.1 Lemborexant	1	723	-14.28 (-33.95 to 5.38)	0.07	0
2.2 Suvorexant	1	111	-10.20 (-15.70 to -4.70)	0.0003	N/A
3 sSE	1	697	4.61 (3.85–5.37)	0.008	0
3.1 Lemborexant	1	697	4.61 (3.85–5.37)	0.008	0
4 sWASO	1	723	-15.08 (-45.57 to 15.41)	0.1	0
4.1 Lemborexant	1	723	-15.08 (-45.57 to 15.41)	0.1	0
5 IDSIQ	1	677	-5.51 (-13.25 to 2.22)	0.09	17
5.1 Daridorexant	1	677	-5.51 (-13.25 to 2.22)	0.09	17
Month 12					
1 sTST	2	1,122	16.38 (0.08–32.68)	0.05	49
1.1 Daridorexant	1	677	10.66 (-5.04 to 26.36)	0.1	0
1.2 Suvorexant	1	445	27.50 (16.30–38.70)	< 0.00001	N/A
2 sTSO	1	445	-9.60 (-16.38 to -2.82)	0.005	N/A
2.1 Suvorexant	1	445	-9.60 (-16.38 to -2.82)	0.005	N/A
3 sWASO	1	445	-9.70 (-16.37 to -3.03)	0.004	N/A
3.1 Suvorexant	1	445	-9.70 (-16.37 to -3.03)	0.004	N/A
4 IDSIQ	1	677	-6.37 (-12.31 to -0.43)	0.04	0
4.1 Daridorexant	1	677	-6.37 (-12.31 to -0.43)	0.04	0

Abbreviations: IDSIQ, Insomnia Daytime Symptoms and Impacts Questionnaire; MD, mean difference; N/A, not availvle; sTST, subjective total sleep time; sTSO, subjective time to sleep onset, sWASO, subjective wake after sleep onset; sSE, subjective sleep efficiency.

At month 6, the pooled analysis of DORAs demonstrated significant improvements in sTST (MD = 19.87; 95%CI: 14.15–25.60; I ^2^ = 0%), sTSO (MD = -12.69; 95%CI: -19.78 to -5.60; I ^2^ = 0%), and sSE (MD = 4.61; 95%CI: 3.85–5.37; I ^2^ = 0%) compared with placebo ( Figure 1 , **Supplementary Material Figures S3,S4** [online only]). No significant differences were observed for sWASO (MD = -15.08; 95%CI: -45.57 to 15.41), nor for the IDSIQ score (MD = -5.51; 95%CI: -13.25 to 2.22) ( **Supplementary Material Figures S5,S6** [online only]).

Relative to placebo, DAR significantly improved the sTST (MD = 14.37; 95%CI: 1.19–27.56), SUV improved the sTST (MD = 24.30; 95%CI: 15.89–32.71) and the sTSO (MD = -10.20; 95%CI: -15.70 to -4.70), and LEM improved the sSE (MD = 4.61; 95%CI: 3.85–5.37). And although the overall effect for sTST, sTSO and sWASO was not significant, LEM 5 mg and 10 mg individually improved those outcomes compared with placebo. The 40-mg dose of SUV also significantly improved the sTST and sTSO, and DAR 50 mg showed significant benefits for sTST and the IDSIQ score ( **Supplementary Material Figures S3** –S6 [online only]).

**Figure 2:**
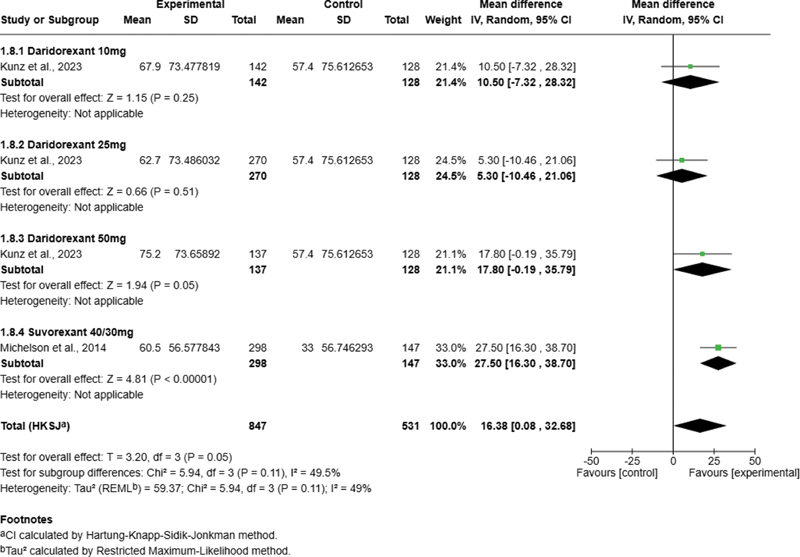
Efficacy of different dosages between the DORA groups and the placebo group at 12 months: sTST.

At 12 months, significant improvements were observed for the sTST (MD = 16.38; 95%CI: 0.08–32.68), sTSO (MD = -9.60; 95%CI: -16.38 to -2.82), sWASO (MD = -9.70; 95%CI: -16.37 to -3.03), and the IDSIQ score (MD = -6.37; 95%CI: -12.31 to -0.43), although moderate heterogeneity was observed (I ^2^ = 49%) for the sTST. No comparisons for the sSE were found for this time point. Daridorexant considerably improved the IDSIQ score (MD = -6.37; 95%CI: -12.31 to -0.43), while SUV improved the sTST (MD = 27.50; 95%CI: 16.30–8.70), the sTSO (MD = -9.60; 95%CI: -16.38 to -2.82) and the sWASO (MD = -9.70; 95%CI: -16.37 to -3.03). Individually, SUV 40/30 mg improved the sTST, sTSO and sWASO, and DAR 50 mg improved the IDSIQ score ( Figure 2 , **Supplementary Material Figure S7** –S9 [online only]).

### Safety

**Figure 3:**
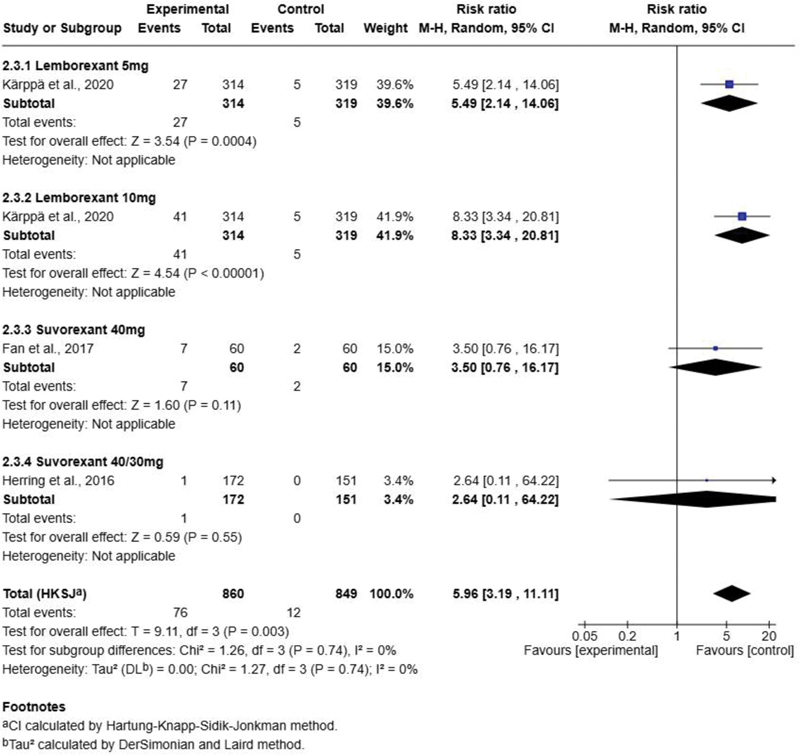
Subanalysis of specific adverse effects (AEs) regarding different doses of DORAs and placebo at 6 months: Somnolence.

At month 6, the overall risk of AEs did not differ between DORAs and placebo (RR = 0.98; 95%CI: 0.90–1.08; I ^2^ = 0%), nor did SAEs (RR = 1.79; 95%CI: 0.96–3.33; I ^2^ = 0%). Somnolence was significantly more frequent in the active-drug group (RR = 5.96; 95%CI: 3.19–11.11) ( Figure 3 ). Other commonly reported AEs, such as headache, upper respiratory-tract infection, urinary-tract infection, back pain and fatigue showed no significant differences, and events such as falls or suicidal ideation were negligible ( Tables 2 3 ).

**Table 2:** Summary and detailed effects sizes from the pair-wise meta-analysis of safety outcomes at months 6 and 12; from all trials using random-effects models

Outcome	Number of trials contributing to the meta-analysis	Number of participants contributing to the meta-analysis	RR (95%CI)	*p* -value	I ^2^ (%)
Month 6					
1 AEs	3	1490	0.98 (0.9–1.08)	0.68	0
1.1 Lemborexant	1	947	0.96 (0.81–1.14)	0.21	0
1.2 Suvorexant	2	543	1.11 (0.81–1.53)	0.29	0
2 SAEs	3	1490	1.79 (0.96–3.33)	0.06	0
2.1 Lemborexant	1	947	1.62 (0.33–8.00)	0.16	0
2.2 Suvorexant	2	543	3.11 (0.00–3,302.57)	0.29	0
Month 12					
1 AEs	2	1454	1.09 (1.06–1.13)	0.002	0
1.1 Daridorexant	1	675	1.10 (1.01–1.20)	0.04	0
1.2 Suvorexant	1	779	1.09 (0.98–1.22)	0.11	N/A
2 SAEs	2	1454	1.62 (0.50–5.19)	0.28	46
2.1 Daridorexant	1	675	2.78 (1.76–4.39)	0.01	0
2.2 Suvorexant	1	779	0.79 (0.44–1.42)	0.42	N/A

Abbreviations: AEs, adverse events; N/A, not availvle; RR, risk ratio; SAEs, serious adverse events.

**Table 3:** Summary of the pair-wise meta-analysis of specific adverse events between the orexin receptor antagonist and the placebo groups at months 6 and 12; from all trials using random-effects models, in which at least 2 trials provided data that could be included

Outcome	Number of trials contributing to the meta-analysis	Number of participants contributing to the meta-analysis	Risk ratio (95%CI)	*p* -value	I ^2^ (%)
Month 6					
1 Somnolence	3	1,390	5.96 (3.19–11.11)	0.003	0
2 Headache	2	1,067	1.20 (0.78–1.84)	0.22	0
3 Upper respiratory tract infection	2	1,370	1.23 (0.66–2.28)	0.37	0
4 Urinary tract infection	2	1,365	0.70 (0.19–2.67)	0.46	15
5 Back pain	2	1,370	1.11 (0.40–3.09)	0.77	4
6 Fatigue	2	1,067	7.26 (0.67–78.72)	0.07	0
Month 12					
1 Somnolence	2	1,454	4.85 (2.52–9.33)	0.009	0
2 Falls	2	1,454	0.93 (0.55–1.59)	0.7	0
3 Excessive daytime sleepiness	2	1,454	2.79 (0.06–138.65)	0.19	0
4 Suicidal ideation	2	1,454	0.56 (0.05–6.52)	0.51	0

At 12 months, the overall AEs were significantly more frequent with DORAs compared with placebo (RR = 1.09; 95%CI: 1.06–1.13; I ^2^ = 0%), but not for SAEs (RR = 1.62; 95%CI: 0.59–5.19). Daridorexant was associated with significantly more AEs and SAEs (RR = 1.10; 95%CI: 1.01–1.20; RR = 2.78; 95%CI: 1.76–4.39 respectively), although analyses stratified by individual doses of DAR and SUV did not show significant differences from placebo. Somnolence remained consistently more common in the active-drug group (RR = 4.85; 95%CI: 2.52–9.33]). No significant differences were observed for SAEs, nor for other specific AEs ( Tables 2 3 ).

### Tolerability

Overall, AEs leading to discontinuation at month 6 did not differ significantly (RR = 1.94; 95%CI: 0.59–6.41; I ^2^ = 53%). However, discontinuation rates were higher for LEM 10 mg (RR = 2.20; 95%CI: 1.13–4.28) and SUV 40/30 mg (RR = 9.22; 95%CI: 2.20–38.67) compared with placebo ( **Supplementary Material Figure S27** [online only]). At month 12, no significant differences in discontinuation were observed between DORAs and placebo overall (RR = 1.06; 95%CI: 0.45–2.51; I ^2^ = 27%), nor for any individual DAR or SUV dose ( Table 4 ).

**Table 4:** Summary and effects sizes from the pair-wise meta-analysis of tolerability outcomes at monthd 6 and 12; from all trials using random-effects models

Outcome	Number of trials contributing to the meta-analysis	Number of participants contributing to the meta-analysis	Risk ratio (95%CI)	*p* -value	I ^2^ (%)
Month 6					
1 Tolerability	3	1,490	1.94 (0.59–6.41)	0.2	53
1.1 Lemborexant	1	947	1.60 (0.02–128.98)	0.4	44
1.2 Suvorexant	2	543	2.23 (0.04–128.04)	0.48	62
Month 12					
1 Tolerability	2	1,462	1.06 (0.45–2.51)	0.84	27
1.1 Daridorexant	1	681	0.83 (0.15–4.59)	0.69	15
1.2 Suvorexant	1	781	1.06 (0.45–2.51)	0.84	27

### League tables

Suvorexant 40/30 mg showed clear superiority over placebo and DAR 25 mg for sTST improvement at month 12, while no other comparisons reached statistical significance ( **Supplementary Material Table S4** [online only]). Overall, there were no statistically significant differences between AEs and SAEs and for tolerability among different dosages at both time points ( **Supplementary Material Tables S5** –S7 [online only]).

## DISCUSSION

The present study included 6 articles, 11 24 25 26 27 28 one being the study by Yardley et. al., 24 which was the second phase of the SUNRISE 2 study, first reported by Kärppä et al., 25 and it included patients that agreed to proceed to a second phase from the study. This second phase rerandomized patients from the placebo group to the experimental groups, LEM5 and LEM10. Thus, there was no placebo control in the second phase, and the study could not be included for placebo comparison in the boxplots and in the league tables for indirect comparison via network metanalysis. In any case, the study by Yardley et. al. 24 presented sustained benefits of LEM5 and 10 over placebo across 12 months. The AEs were mostly mild to moderate, and the most common were nasopharyngitis, somnolence, and headache. Those results are consistent with proportions found among studies included in the current metanalysis.

Previous systematic reviews 29 30 31 32 that evaluated earlier time points using subjective and objective outcomes have already demonstrated improvements in sleep parameters. Among patients treated with DORAs, the present meta-analysis identified maintained improvements in efficacy outcomes at 6 months, such as sTST, sTSO, and sSE. In contrast, sWASO and the IDSIQ score showed no significant benefits at this time point, and wide confidence intervals were observed for the overall sample. In month 12, there were significant improvements for sTST, sTSO, sWASO, and the IDSIQ score. Thus, DORAs showed sustained efficacy for as long as 12 months. However, it is important to note that the sTSO and sWASO estimates at this time point were derived only from the SUV 40/30 mg trial by Michelson et al., 26 which limits the generalizability of these findings to other DORAs. The substantial heterogeneity observed for sTST in month 12 may be partly due to small sample sizes and variability in effect sizes between lower and higher doses.

Regarding the safety profile in month 6, no statistically significant differences were observed between DORAs and placebo in the overall AEs or SAEs. In month 12, DORAs were associated with a significantly-increased risk of AEs compared with placebo. Somnolence occurred significantly more frequently than with placebo at both time points. Fatigue was another notable AE, particularly for LEM, which demonstrated a dose-dependent increase in fatigue in month 6. Other specific AEs, such as headache, nasopharyngitis, upper respiratory-tract infections, urinary-tract infections, back pain, falls, suicidal ideation, and excessive daytime sleepiness did not differ significantly from placebo, neither individually, nor in the pooled analysis.

Traditional hypnotics, such as benzodiazepines and Z-drugs, are linked to the development of tolerance, physiological dependence, and rebound insomnia upon discontinuation. 33 Furthermore, these drugs carry a well-documented, 34 35 dose-dependent risk of next-day cognitive and psychomotor impairment, which significantly increases the risk of falls, hip fractures, and motor vehicle accidents, particularly in older adults. In contrast, we found here a lack of such major AEs and a safety profile comparable to that of placebo, suggesting that DORAs exhibit a superior long-term safety and tolerability profiles compared with Z-drugs, 34 although somnolence remained consistently more frequent at both time points.

Finally, we did not find statistically-significant differences for AEs leading to discontinuation between interventions and placebo in months 6 and 12. However, LEM 10 mg and SUV 40/30 mg did present statistically-significant differences, favoring the control group in month 6. Xue et al. 30 noticed that higher doses of SUV for long-term use were associated with more AEs, which might reflect the discontinuation found here, although discontinuation for SUV 40/30 mg was not significant in month 12. Higher doses of DORAs tended to increase discontinuation, but there was no statistically-significant difference. Moreover, we found an important heterogeneity, which could be due to differences among doses.

Suvorexant (Belsomra; Merck Sharp & Dohme LLC) was the first DORA approved for primary insomnia, receiving United States FDA approval in 2014, followed by countries such as Japan, Canada, and Australia. 36 Suvorexant showed significant results for sTST, sTSO and sWASO values. Suvorexant 40/30mg presented the greatest increases for sTST in months 6 and 12. This same dose was also associated with most overall AEs and SAEs in month 6, but this trend was not observed in month 12, although somnolence was significant at this time point. Consistently, Xue et al. 30 also found great efficacy for SUV and as higher risk of AEs for higher doses. In the United States and Japan, the highest approved dose for SUV is 20 mg. 37

Lemborexant (Dayvigo; Eisai Inc.) was approved for the treatment of insomnia in the United States in 2019, with subsequent approvals in Japan, Canada, Australia, and other Asian countries. 36 38 The 5- and 10-mg doses of LEM significantly improved the sTST, sTSO and sSE levels in a dose-dependent manner. Similar to the findings by Xue et al., 29 30 in month 6, LEM did not increase the overall AEs and SAEs in the current study. However, individual LEM doses significantly increased somnolence and fatigue outcomes. The 10-mg dose also showed significantly more AEs leading to discontinuation in month 6 compared with placebo.

Daridorexant (Quviviq; Idorsia Pharmaceuticals) is the most recently-approved DORA, receiving FDA approval in 2022 at doses of 25 mg and 50 mg for the treatment of insomnia, and subsequently approved in Europe, Canada, and Japan. 39 In the present analysis, DAR 10 and 25 mg did not show significant results for the sTST and the IDSIQ score, while DAR 50 mg did improve those outcomes. Rocha et al. 31 and Xue et al. 30 did find improvements for some outcomes, but greater improvements were usually observed for DAR 50 mg. Those findings suggest that higher doses of DAR might have greater benefits for insomnia. Daridorexant also tended to show a dose-dependent increase in AEs, SAEs, somnolence and AEs leading to discontinuation in month 12. Daridorexant 50 mg was worse tolerated than DAR 25 mg and SUV 40/30 mg, and significantly worse than DAR 10 mg.

### Limitations

The major limitation of the current study is the very-limited data, since DORAs are a relatively-new class of drug for insomnia treatment. Additionally, the study sample only included adults with no further psychiatric comorbidities. The present study analyzed only 6 articles, 11 24 25 26 27 28 with a limited population sample for the overall class, thus compromising the generalizability of the results. Second, we found considerable heterogeneity for sTST and tolerability in month 6. Our analysis included studies with reasonably-similar methodologies, but differences could influence the results found and must not be disregarded.

In conclusion, the current study found that DORAs generally improve sleep parameters, while remaining tolerable, although somnolence was consistently reported at both time points. Since DORAs are a relatively-new class of drugs, the number of RCTs are limited, especially for longer periods of time. More studies with similar doses and long-term follow-up are needed to increase our confidence in what are the real effects of DORAs on long-term sleep parameters, as well as their safety and tolerability profiles. Some DORAs, such as filorexant and almorexant, have not yet been approved by the FDA, and larger studies of approved doses are still needed, as knowledge about this class increases.
